# Giant mature teratoma of the retroperitoneal pelvic floor in an adult with combined developmental malformations of the reproductive system: A case report

**DOI:** 10.1097/MD.0000000000042889

**Published:** 2025-06-13

**Authors:** Zeyang He, Yufei Zeng, Xuhua Chen, Huiying Wang

**Affiliations:** a Department of Gynecology, Shenzhen Hospital, Beijing University of Chinese Medicine, Shenzhen, China.

**Keywords:** pelvic floor tumor, retroperitoneal tumor, teratoma

## Abstract

**Rationale:**

Teratoma is a germ cell tumor with multidirectional differentiation potential, but retroperitoneal and sacrococcygeal teratomas are relatively rare.

**Patient concerns:**

A 32-year-old adult woman with a right duplicated ureter and left unicornuate uterus was found to have had a large pelvic mass for more than 8 years, which was increasing in size year by year.

**Diagnoses:**

After 2 surgeries that failed to remove the pelvic mass completely, and the biopsy of the mass only suggested that it was a mature teratoma.

**Interventions:**

The third time, after the transabdominal and transsacral combined pathway surgery, the mass was finally removed completely and found to have grown from the pelvic floor to the presacral area and finally to the right pelvic wall.

**Outcomes:**

At 1-year follow-up, the patient had no complications, and serial pelvic ultrasounds showed no recurrence or metastasis.

**Lessons:**

Thus, we demonstrate the feasibility of a combined transabdominal and transsacral approach for resecting this large mature retroperitoneal teratoma of the pelvic floor, providing a reference for surgeons managing similarly complex cases.

## 1. Introduction

Teratoma is a germ cell tumor with multidirectional differentiation potential originating from 2 or more germ layers, which is mostly benign and occurs in gonads such as ovaries and testes, etc. Teratomas occurring in the retroperitoneum and sacrococcygeal region are less common, accounting for only 4% of all primary teratomas, and the corresponding clinical reports are also less frequent.^[1]^ We report a rare case of a woman of childbearing age with a combination of a giant mature teratoma of the retroperitoneal pelvic floor.

## 2. Case presentation

A 32-year-old adult woman with a combination of a right duplicated lower ureter and a left unicornuate uterus had a history of 2 cesarean sections, bilateral hydronephrosis, and no other underlying diseases. Eight years ago, a gynecologic ultrasound found a mixed mass on the right ovary measuring about 52 mm × 50 mm × 35 mm, and in the first operation for right tubal reimplantation, found that the pelvic mass was clearly encapsulated by blood vessels, and did not do further exploration, but only took a pathologic biopsy. Five years ago, a gynecological ultrasound was performed and found that a mass measuring about 78 mm × 50 mm was seen in the right adnexal region, with mixed echoes, dense small light spots, a slightly thicker cystic wall, and clear borders, so teratoma was considered a possibility. The second laparoscopic exploration revealed a slightly protruding cystic mass in the retroperitoneal right pelvis near the obturator nerve region, with abundant peripheral blood vessels and nerves, and the mass could be detected by anal palpation. During the operation, the laparoscopic surgery was found to be difficult, and was transferred to open surgery, but it was found that the difficulty and risk of the operation were greater, multidisciplinary collaborative surgery was required, and the cyst was only incised to close the wall of the cyst after removing as much of the contents as possible. Biopsy pathology suggests pelvic wall mature teratoma. The patient’s gynecological ultrasound was repeated this year, suggesting that there were multiple space-occupying lesions in the right adnexal region, and teratoma was considered a possibility (sizes of 66 mm × 47 mm, 57 mm × 36 mm, 29 mm × 19mm, and 18 mm × 13 mm, respectively), and she was again recommended to be admitted to the hospital for surgical treatment.

## 3. Investigations

**Figure 1.**
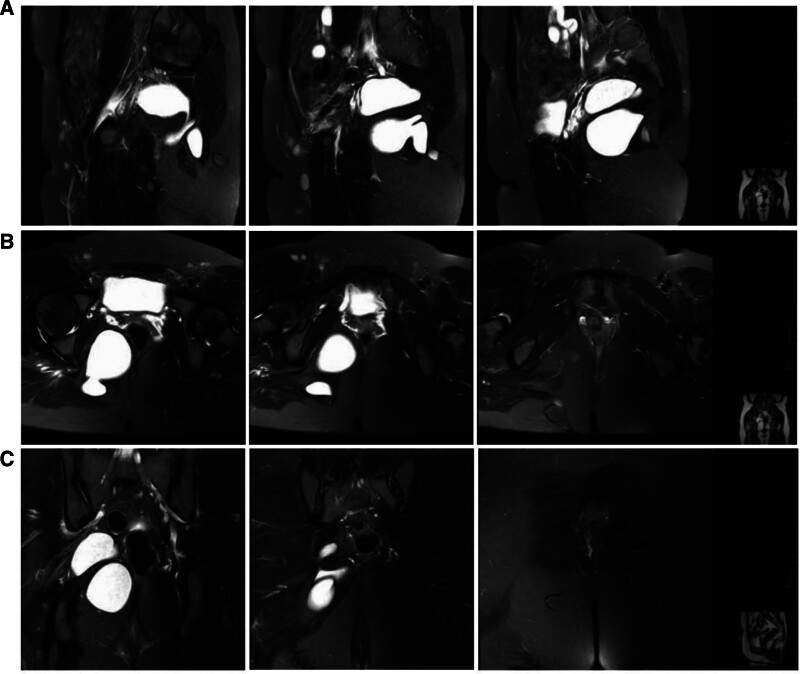
MR images of a retroperitoneal pelvic floor teratoma: sagittal view (A), cross-sectional view (B), and coronal view (C). MR = magnetic resonance.

After admission to the hospital, vagino-recto-abdominal examination was performed, and a mass measuring about 70 mm × 60 mm was found in the right adnexal region, and a mass measuring about 30 mm × 20 mm was found on the right side of the sacrococcygeal region, which was hard and had poor mobility, and there was an indentation on the right side of the buttock near the sacrum, with no obvious skin lesions, and the patient complained that the indentation had once had a secretion. Magnetic resonance examination revealed irregular cystic signal foci in the soft tissues of the right pelvic region and the posterior gluteal region, with a size of about 88 mm × 65 mm × 52 mm. The uppermost part of the foci was at the level of the sacral 4 vertebrae, and the lowermost part was located at the level of the inferior gemellus muscle, with a small portion of the foci entering into the gluteus maximus muscle and extending subcutaneously, which was considered to be cystic occupancy in the soft tissues of the right pelvic region and the posterior gluteal region and was considered to be benign with the formation of fistulae (Fig. 1).

## 4. Treatment

**Figure 2.**
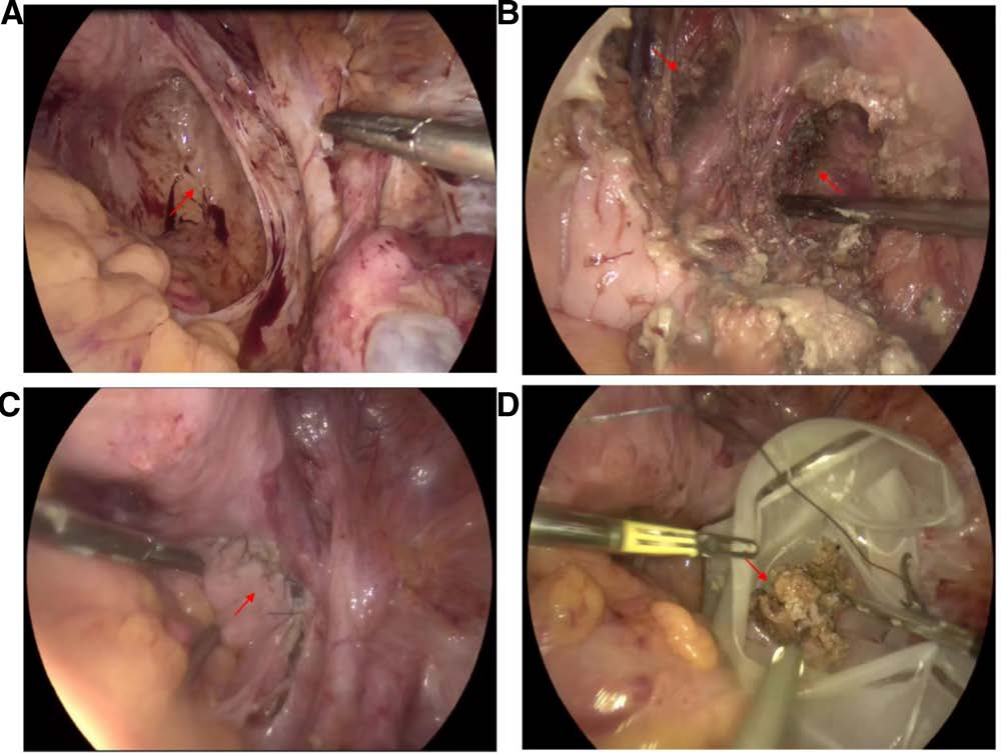
We show the position of the attached retroperitoneal pelvic floor teratoma (arrow) before laparoscopic unresection (A). We show the retroperitoneal pelvic floor teratoma after resection and the 2 sides of the tunnel (arrows) (B). Localized suture (arrow) (C). The capsule of the removed teratoma in specimen bag (arrow) (D).

After a multidisciplinary consultation, it was decided to remove part of the cyst from the buttock to loosen the surrounding tissues of the cyst, and then remove the mass completely from the pelvis through laparoscopy. During the operation, the patient was first allowed to take the folding knife position, and a transverse fusiform incision was made along the skin surface of the right buttock depression, and a mass was seen after incising the skin and subcutaneous tissues layer by layer, and the cyst wall was carefully peeled away from the interstitial space of the surrounding tissues, and the mass was completely peeled off, and another mass was seen in the vicinity of the deeper layer, which was continued to be carefully separated, and found to have passed through the levator ani, and most of which was located in the interstitial space of the levator ani, and it was tried to release the mass and the surrounding tissues as much as possible. When it cannot be separated, use No. 1 silk thread to ligate the tumor tissue markers at that location, and suture the external fascia of the levator ani muscle and each layer of the incision . The patient was changed to the lithotomy position, and the laparoscopic exploration revealed the following: mild adhesion between the intestinal canal and the abdominal wall, left unicornuate uterus, mild membranous adhesion between the left ovary, fallopian tube and the intestinal canal, the right ovary was isolated on the surface of the common iliac artery and part of the right fallopian tube was seen, and a cystic mass protruded from the retroperitoneal area of the right pelvic wall close to the obturator nerve region; the surface was flaky, old scar tissue. The thickened ureter was seen crossing the anterolateral side of the tumor and reaching the upper outer part of the bladder. The peritoneum was opened from the right uterosacral ligament, and the mass was found to be densely adherent to the surrounding tissues, with obvious scarred hyperplastic tissues visible. Sharp separation of the surrounding tissues of the right pelvic wall from the interstitial space of the mass was carried out with care for the surrounding blood vessels and nerves, and the incision was enlarged to peel off the cyst; during the process of separation, the wall of the capsule tore, and a fat-like fluid accompanied with a small amount of hair flowed out, and after the suction of all the fat and grease was completed, it was found that the mass seemed to communicate with the gluteal mass; after the separation of the right levator ani muscle, another mass was seen. Then carefully separating the mass from the surrounding muscle tissue, the previous ligature was seen. Continuing to separate along the mass, it was found to be connected to the right pelvic wall mass, and after disconnecting the mass, the mass was completely removed (Fig. 2).

## 5. Outcome and follow-up

During 1 year of follow-up after discharge, the patient experienced no surgical complications. Serial pelvic ultrasounds showed no evidence of tumor recurrence or metastasis.

## 6. Discussion

Primary retroperitoneal teratomas account for about 1% to 11% of primary retroperitoneal tumors,^[1]^ commonly occurring in infants and children,^[2]^ with only 10% to 20% occurring in adults over 30 years of age.^[3]^ Therefore, a primary retroperitoneal combined with a giant teratoma of the pelvic floor is very rare in this patient.

Primary pelvic retroperitoneal tumors are usually hidden and deep in location, so there are no obvious symptoms in the early stage and lack of specific clinical manifestations, and a large part of them are found accidentally through pelvic imaging examination. However, when the tumor increases to a certain degree, it can produce obvious compression symptoms, such as bladder compression can cause urinary urgency, urinary difficulty or even urinary retention, ureteral compression can lead to oliguria or even pyelonephrosis,^[4]^ rectal compression can cause defecation difficulty, or even constipation,^[5]^ and presacral nerve compression can lead to pain and numbness in the buttocks or lower limbs. In this case, the patient had no specific clinical manifestations, and the gynecological ultrasound examination revealed that the tumor was located in the retroperitoneum on the right side of the pelvic wall extending to the pelvic floor, and the patient had bilateral hydronephrosis, which could not be ruled out as a result of the compression by the mass before the operation.

The treatment strategies for primary pelvic retroperitoneal tumors include drug and surgical treatments, with surgery being the main treatment method.^[6]^ Due to the complexity of pelvic retroperitoneal anatomical structures, the type and scope of surgery vary greatly accordingly, and the surgery is more difficult, and multidisciplinary assistance for the surgery may often be required, so the pathology specimen appears to be of vital importance.^[7]^ When the lesion cannot be removed or the patient refuses surgery, an ultrasound or computerized tomography-guided puncture biopsy can be performed to clarify the type of pathology and then choose the next diagnostic and treatment plan.^[8]^ In this case, the patient found it difficult to remove the lesion completely in the first 2 surgeries, and the risk of surgery was high, so a pathologic biopsy was performed.

Surgical approaches for primary pelvic retroperitoneal tumors should be decided based on the actual situation such as tumor size, location, and nature. It is generally divided into transabdominal, transsacral, and combined transabdominal and transsacral pathways. If the lower pole of the tumor has not breached the levator ani, and the 4th and 5th sacral vertebrae and coccyx have not been invaded, transabdominal resection can be performed; if the upper pole of the tumor is located below the level of the 3rd sacral vertebra and it is expected that the upper pole is easy to be separated, transsacral route can be chosen; if the tumor is huge and exceeds the above-mentioned conditions, then transabdominal and transsacral combined routes are more often needed. Choosing the appropriate surgical approach can improve the tumor resection rate and reduce side injuries and postoperative complications. In this case, the patient failed to choose the appropriate surgical approach in the second operation and chose the transabdominal route alone, which led to the difficulty of tumor resection. The patient’s lesion reached down to the levator ani and extended to the subcutaneously layer to form a fistula. The upper edge is approximately at the level of the 4th sacral vertebra and should be resected via the sacral region . And the patient had undergone multiple surgeries and had abnormalities in the development of the genitourinary system, and the abdominal cavity was complicated, which should be resected via the abdomen. Moreover, most of the lesions in this patient were located in the right pelvic wall, which was deeper, and if open surgery was chosen, the surgical field of vision and operating space were limited, so laparoscopic tumor resection was selected. So the third surgical approach was chosen after a full evaluation Transabdominal Transsacral Combined Path. The patient was allowed to take the folding knife position to isolate the mass as much as possible via the sacrum, and then take the lithotomy position to perform laparoscopic mass resection. Thus, the combined abdominal-transsacral approach requires thorough preoperative evaluation and careful surgical selection, demanding extensive anatomical knowledge and advanced surgical skills. Potential risks include injury to adjacent organs such as intestines, bladder, or ureters. We, therefore, recommend multidisciplinary preoperative planning involving general surgery, urology, colorectal surgery, orthopedics, and neurosurgery to optimize surgical outcomes.

In terms of prognosis, mature teratomas generally have a favorable prognosis, with a 5-year survival rate approaching 100% after treatment.^[9]^ Nevertheless, close follow-up is still recommended because the incidence of malignancy is about 3% to 6%.^[10]^ In this case, the patient was followed up for 1 year after surgery with no signs of recurrence.

From this case, we demonstrate the feasibility of a combined transabdominal and transsacral approach for resecting this large mature retroperitoneal teratoma of the pelvic floor, providing a reference for surgeons managing similarly complex cases.

## Author contributions

**Writing – original draft:** Zeyang He.

**Writing – review & editing:** Zeyang He, Huiying Wang.

**Resources:** Yufei Zeng, Xuhua Chen.

**Supervision:** Yufei Zeng, Huiying Wang.

## References

[1] Sasi W Ricchetti GA Parvanta L Carpenter R. Giant mature primary retroperitoneal teratoma in a young adult: report of a rare case and literature review. Case Rep Surg. 2014;2014:930538. 25506459 10.1155/2014/930538 PMC4258356

[2] Luo CC Huang CS Chu SM Chao HC Yang CP Hsueh C. Retroperitoneal teratomas in infancy and childhood. Pediatr Surg Int. 2005;21:536–40. 15918045 10.1007/s00383-005-1424-7

[3] Panageas E. General diagnosis case of the day. Primary retroperitoneal teratoma. AJR Am J Roentgenol. 1991;156:1292–4. 2028883 10.2214/ajr.156.6.2028883

[4] Gatcombe HG Assikis V Kooby D Johnstone PA. Primary retroperitoneal teratomas: a review of the literature. J Surg Oncol. 2004;86:107–13. 15112254 10.1002/jso.20043

[5] Ratan SK Ratan J Kalra R. Large benign cystic teratoma of the mesosigmoid causing intestinal obstruction: report of a case. Surg Today. 2002;32:922–4. 12376796 10.1007/s005950200183

[6] Singh C Raypattanaik NM Sharma I Kaman L. Primary retroperitoneal teratoma in a young male: a case report. Cureus. 2021;13:e15376. 34249529 10.7759/cureus.15376 PMC8248951

[7] Huang X Liu B Xie L. Giant primary retroperitoneal teratoma in an adult female patient: a case report. Oncol Lett. 2013;6:460–2. 24137347 10.3892/ol.2013.1374 PMC3789054

[8] Yoon SS Tanabe KK Warshaw AL. Adult primary retroperitoneal teratoma. Surgery. 2005;137:663–4. 15933637 10.1016/j.surg.2004.02.002

[9] Pinson CW ReMine SG Fletcher WS Braasch JW. Long-term results with primary retroperitoneal tumors. Arch Surg. 1989;124:1168–73. 2802979 10.1001/archsurg.1989.01410100070012

[10] Sato F Mimata H Mori K. Primary retroperitoneal mature cystic teratoma presenting as an adrenal tumor in an adult. Int J Urol. 2010;17:817. 20636475 10.1111/j.1442-2042.2010.02591.x

